# Diaqua­(trifluoro­acetato-κ^2^ *O*,*O*′)[2,4,6-tri-2-pyridyl-1,3,5-triazine-κ^3^ *N* ^2^,*N* ^1^,*N* ^6^]manganese(II) trifluoro­acetate

**DOI:** 10.1107/S1600536809015098

**Published:** 2009-04-30

**Authors:** Kong Mun Lo, Seik Weng Ng

**Affiliations:** a Department of Chemistry, University of Malaya, 50603 Kuala Lumpur, Malaysia

## Abstract

The Mn^II^ atom in the two independent ion-pairs of the title salt, [Mn(C_2_F_3_O_2_)(C_18_H_12_N_6_)(H_2_O)_2_]C_2_F_3_O_2_, is *N*,*N*′,*N*′′-chelated by the neutral *N*-heterocycle and *O*,*O*′-chelated by the carboxyl­ate ion, the five atoms involved in chelation comprising a penta­gon around it. The apical sites of the *trans*-penta­gonal bipyramidal coordination geometry are occupied by two water mol­ecules. The cations and lattice anions are linked by O—H⋯O and O—H⋯N hydrogen bonds into a three-dimensional network.

## Related literature

For the seven-coordinate diaqua­(acetato)[2,4,6-tris­(2-pyrid­yl)-1,3,5-triazine)]manganese(II) cation, see: Majumder *et al.* (2005 ▶); Zhao *et al.* (2007 ▶). For the synthesis of manganese trifluoro­acetate, see: Baillie *et al.* (1968 ▶). The compound can be synthesized directly from manganese metal and trifluoro­acetic acid; see: Hübner *et al.* (2006 ▶).
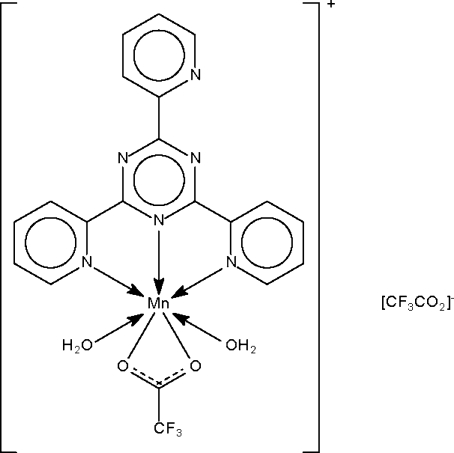


## Experimental

### 

#### Crystal data

[Mn(C_2_F_3_O_2_)(C_18_H_12_N_6_)(H_2_O)_2_]C_2_F_3_O_2_
*M* *_r_* = 629.35
Monoclinic, 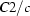

*a* = 31.1179 (4) Å
*b* = 10.5881 (1) Å
*c* = 32.9673 (4) Å
β = 117.027 (1)°
*V* = 9675.8 (2) Å^3^
*Z* = 16
Mo *K*α radiation
μ = 0.65 mm^−1^
*T* = 100 K
0.40 × 0.30 × 0.20 mm

#### Data collection

Bruker SMART APEX diffractometer
Absorption correction: multi-scan (*SADABS*; Sheldrick, 1996 ▶) *T*_min_ = 0.782, *T*_max_ = 0.882
58407 measured reflections
11090 independent reflections
8998 reflections with *I* > 2σ(*I*)
*R*_int_ = 0.035

#### Refinement

*R*[*F* ^2^ > 2σ(*F* ^2^)] = 0.038
*wR*(*F* ^2^) = 0.113
*S* = 1.26
11090 reflections
763 parameters
12 restraints
H atoms treated by a mixture of independent and constrained refinement
Δρ_max_ = 0.70 e Å^−3^
Δρ_min_ = −0.45 e Å^−3^

### 

Data collection: *APEX2* (Bruker, 2007 ▶); cell refinement: *SAINT* (Bruker, 2007 ▶); data reduction: *SAINT*; program(s) used to solve structure: *SHELXS97* (Sheldrick, 2008 ▶); program(s) used to refine structure: *SHELXL97* (Sheldrick, 2008 ▶); molecular graphics: *X-SEED* (Barbour, 2001 ▶); software used to prepare material for publication: *publCIF* (Westrip, 2009 ▶).

## Supplementary Material

Crystal structure: contains datablocks global, I. DOI: 10.1107/S1600536809015098/xu2515sup1.cif

Structure factors: contains datablocks I. DOI: 10.1107/S1600536809015098/xu2515Isup2.hkl

Additional supplementary materials:  crystallographic information; 3D view; checkCIF report

## Figures and Tables

**Table 1:** Selected geometric parameters (Å, °)

Mn1—O1	2.301 (2)
Mn1—O2	2.396 (2)
Mn1—O1*w*	2.119 (2)
Mn1—O2*w*	2.146 (2)
Mn1—N1	2.389 (2)
Mn1—N2	2.261 (2)
Mn1—N3	2.411 (2)
Mn2—O3	2.440 (2)
Mn2—O4	2.293 (2)
Mn2—O3*w*	2.127 (2)
Mn2—O4*w*	2.135 (2)
Mn2—N7	2.400 (2)
Mn2—N8	2.279 (2)
Mn2—N9	2.386 (2)

**Table 2:** Hydrogen-bond geometry (Å, °)

*D*—H⋯*A*	*D*—H	H⋯*A*	*D*⋯*A*	*D*—H⋯*A*
O1*w*—H1*w*1⋯O5	0.84 (3)	1.82 (3)	2.661 (2)	176 (3)
O1*w*—H1*w*2⋯N12	0.84 (3)	1.94 (3)	2.765 (2)	166 (3)
O2*w*—H2*w*1⋯O2^i^	0.84 (3)	1.96 (3)	2.789 (2)	169 (3)
O2*w*—H2*w*2⋯O8^ii^	0.84 (3)	1.85 (3)	2.679 (2)	167 (3)
O3*w*—H3*w*1⋯N6	0.84 (3)	1.98 (3)	2.798 (2)	167 (2)
O3*w*—H3*w*2⋯O7	0.84 (3)	1.86 (3)	2.703 (2)	175 (3)
O4*w*—H4*w*1⋯O3^iii^	0.84 (3)	1.95 (3)	2.782 (2)	170 (3)
O4*w*—H4*w*2⋯O6^iv^	0.84 (3)	1.87 (3)	2.672 (2)	160 (2)

Symmetry codes: (i) 
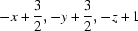
; (ii) 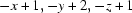; (iii) 
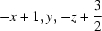
; (iv) 
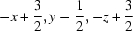
.

**Fig. 1.**
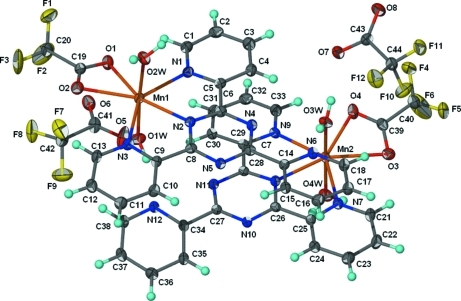
70% Probability thermal ellipsoid plot of the two independent ion-pairs of [Mn(H2O)2(C18H12N6)(C2F3O2)] [C2F3O2]. Hydrogen atoms are drawn as spheres of arbitrary radius.

## supplementary crystallographic information

### Experimental

Manganese trifluoroacetate was synthesized *in situ* by using a procedure slightly different from the reported one (Baillie *et al.*, 1968). Manganese acetate tetrahydrate (0.7 g, 3 mmol) was dissolved in ethanol (100 ml) along with trifluoroacetic acid (0.3 ml, 3.2 mmol). The solution was heated until acetate dissolved completely; commercially available 2,4,6-tris(2-pyridyl)-1,3,5-triazine (0.9 g, 3 mmol) was added. Slow cooling of the solution yielded deep yellow crystals.

### Refinement

Carbon-bound H-atoms were placed in calculated positions (C—H 0.95 Å) and were included in the refinement in the riding model approximation, with *U*(H) set to 1.2*U*(C). The oxygen-bound H-atom were located in a difference Fourier map, and were refined with a distance restraint of O–H 0.84±0.01 Å; their temperature factors were tied by a factor of 1.5

### Crystal data

**Table:**

[Mn(C_2_F_3_O_2_)(C_18_H_12_N_6_)(H_2_O)_2_]C_2_F_3_O_2_	*F*(000) = 5072
*M**_r_* = 629.35	*D*_x_ = 1.728 Mg m^−^^3^
Monoclinic, *C*2/*c*	Mo *K*α radiation, λ = 0.71073 Å
Hall symbol: -C 2yc	Cell parameters from 9826 reflections
*a* = 31.1179 (4) Å	θ = 2.1–28.3°
*b* = 10.5881 (1) Å	µ = 0.65 mm^−^^1^
*c* = 32.9673 (4) Å	*T* = 100 K
β = 117.027 (1)°	Triangular block, deep yellow
*V* = 9675.8 (2) Å^3^	0.40 × 0.30 × 0.20 mm
*Z* = 16	

### Data collection

**Table:**

Bruker SMART APEX diffractometer	11090 independent reflections
Radiation source: fine-focus sealed tube	8998 reflections with *I* > 2σ(*I*)
graphite	*R*_int_ = 0.035
ω scans	θ_max_ = 27.5°, θ_min_ = 1.4°
Absorption correction: multi-scan (*SADABS*; Sheldrick, 1996)	*h* = −40→40
*T*_min_ = 0.782, *T*_max_ = 0.882	*k* = −13→13
58407 measured reflections	*l* = −42→36

### Refinement

**Table:**

Refinement on *F*^2^	Primary atom site location: structure-invariant direct methods
Least-squares matrix: full	Secondary atom site location: difference Fourier map
*R*[*F*^2^ > 2σ(*F*^2^)] = 0.038	Hydrogen site location: inferred from neighbouring sites
*wR*(*F*^2^) = 0.113	H atoms treated by a mixture of independent and constrained refinement
*S* = 1.26	*w* = 1/[σ^2^(*F*_o_^2^) + (0.05*P*)^2^ + 1*P*] where *P* = (*F*_o_^2^ + 2*F*_c_^2^)/3
11090 reflections	(Δ/σ)_max_ = 0.001
763 parameters	Δρ_max_ = 0.70 e Å^−^^3^
12 restraints	Δρ_min_ = −0.45 e Å^−^^3^

### Fractional atomic coordinates and isotropic or equivalent isotropic displacement parameters (Å^2^)

**Table:**

	*x*	*y*	*z*	*U*_iso_*/*U*_eq_	
Mn1	0.720616 (11)	0.77896 (3)	0.571168 (11)	0.01405 (8)	
Mn2	0.543400 (11)	0.73110 (3)	0.683854 (11)	0.01352 (8)	
F1	0.81794 (5)	1.08937 (15)	0.54644 (5)	0.0348 (4)	
F2	0.85747 (6)	1.05565 (19)	0.61763 (6)	0.0511 (5)	
F3	0.86788 (6)	0.93594 (17)	0.57115 (9)	0.0641 (7)	
F4	0.42111 (5)	1.04711 (14)	0.64551 (6)	0.0350 (4)	
F5	0.38758 (5)	0.89407 (16)	0.66211 (7)	0.0424 (4)	
F6	0.43881 (7)	1.0104 (2)	0.71532 (6)	0.0581 (6)	
F7	0.85946 (5)	0.81672 (15)	0.66461 (5)	0.0315 (3)	
F8	0.91840 (5)	0.90901 (15)	0.71976 (6)	0.0363 (4)	
F9	0.88793 (7)	0.74277 (16)	0.73215 (6)	0.0429 (4)	
F10	0.39983 (5)	0.82711 (14)	0.57845 (5)	0.0302 (3)	
F11	0.34953 (4)	0.88917 (13)	0.51150 (5)	0.0259 (3)	
F12	0.40909 (5)	0.76990 (14)	0.52039 (5)	0.0315 (3)	
O1	0.76515 (5)	0.96203 (15)	0.58714 (6)	0.0216 (3)	
O2	0.79144 (5)	0.80310 (14)	0.56061 (5)	0.0193 (3)	
O3	0.46788 (6)	0.75451 (15)	0.68873 (6)	0.0212 (3)	
O4	0.50553 (5)	0.92045 (15)	0.67875 (6)	0.0213 (3)	
O5	0.80460 (6)	0.87181 (18)	0.71467 (6)	0.0326 (4)	
O6	0.85065 (5)	1.04341 (16)	0.72582 (6)	0.0231 (3)	
O7	0.47236 (6)	0.96368 (16)	0.56391 (6)	0.0280 (4)	
O8	0.40676 (6)	1.08581 (15)	0.53004 (6)	0.0272 (4)	
O1w	0.75416 (6)	0.73125 (15)	0.64126 (5)	0.0212 (3)	
H1w1	0.7709 (9)	0.777 (2)	0.6639 (6)	0.032*	
H1w2	0.7481 (10)	0.6617 (14)	0.6497 (8)	0.032*	
O2w	0.68227 (5)	0.81939 (16)	0.49969 (5)	0.0208 (3)	
H2w1	0.6866 (9)	0.777 (2)	0.4803 (7)	0.031*	
H2w2	0.6549 (6)	0.854 (2)	0.4869 (8)	0.031*	
O3w	0.51060 (5)	0.75031 (15)	0.61179 (5)	0.0175 (3)	
H3w1	0.5102 (9)	0.6916 (16)	0.5946 (7)	0.026*	
H3w2	0.4969 (9)	0.8149 (14)	0.5963 (7)	0.026*	
O4w	0.57517 (6)	0.69922 (16)	0.75559 (5)	0.0210 (3)	
H4w1	0.5613 (9)	0.706 (3)	0.7722 (8)	0.032*	
H4w2	0.5966 (8)	0.643 (2)	0.7662 (8)	0.032*	
N1	0.66473 (6)	0.91189 (16)	0.58210 (6)	0.0144 (3)	
N2	0.65646 (6)	0.66536 (16)	0.56512 (6)	0.0137 (3)	
N3	0.73037 (6)	0.55813 (17)	0.55953 (6)	0.0155 (4)	
N4	0.58257 (6)	0.65776 (17)	0.56871 (6)	0.0146 (3)	
N5	0.61673 (6)	0.46946 (17)	0.55619 (6)	0.0140 (3)	
N6	0.50663 (6)	0.52889 (17)	0.56389 (6)	0.0151 (3)	
N7	0.52455 (6)	0.51015 (16)	0.67239 (6)	0.0143 (3)	
N8	0.60343 (6)	0.61949 (16)	0.67885 (6)	0.0132 (3)	
N9	0.61090 (6)	0.86374 (17)	0.69854 (6)	0.0144 (3)	
N10	0.63562 (6)	0.42548 (16)	0.66984 (6)	0.0136 (3)	
N11	0.68154 (6)	0.61471 (16)	0.68407 (6)	0.0143 (3)	
N12	0.74802 (6)	0.48710 (17)	0.66792 (6)	0.0153 (3)	
C1	0.66939 (8)	1.0355 (2)	0.59036 (8)	0.0183 (4)	
H1	0.6980	1.0759	0.5934	0.022*	
C2	0.63460 (8)	1.1087 (2)	0.59474 (8)	0.0194 (4)	
H2	0.6393	1.1968	0.6003	0.023*	
C3	0.59305 (8)	1.0504 (2)	0.59078 (8)	0.0200 (4)	
H3	0.5686	1.0979	0.5935	0.024*	
C4	0.58779 (8)	0.9217 (2)	0.58278 (8)	0.0183 (4)	
H4	0.5598	0.8789	0.5801	0.022*	
C5	0.62422 (7)	0.85697 (19)	0.57883 (7)	0.0142 (4)	
C6	0.62073 (7)	0.71916 (19)	0.57034 (7)	0.0140 (4)	
C7	0.58187 (7)	0.53387 (19)	0.56043 (7)	0.0137 (4)	
C8	0.65341 (7)	0.53987 (19)	0.55900 (7)	0.0135 (4)	
C9	0.69506 (7)	0.47888 (19)	0.55673 (7)	0.0138 (4)	
C10	0.69771 (7)	0.3489 (2)	0.55397 (7)	0.0168 (4)	
H10	0.6718	0.2967	0.5515	0.020*	
C11	0.73901 (8)	0.2963 (2)	0.55483 (8)	0.0193 (4)	
H11	0.7421	0.2074	0.5532	0.023*	
C12	0.77551 (8)	0.3763 (2)	0.55805 (8)	0.0203 (4)	
H12	0.8043	0.3432	0.5590	0.024*	
C13	0.76949 (8)	0.5061 (2)	0.55991 (8)	0.0191 (4)	
H13	0.7946	0.5603	0.5615	0.023*	
C14	0.53838 (7)	0.4630 (2)	0.55499 (7)	0.0139 (4)	
C15	0.53145 (7)	0.3379 (2)	0.54099 (7)	0.0158 (4)	
H15	0.5544	0.2951	0.5345	0.019*	
C16	0.49008 (8)	0.2768 (2)	0.53669 (8)	0.0187 (4)	
H16	0.4845	0.1907	0.5277	0.022*	
C17	0.45715 (7)	0.3436 (2)	0.54574 (7)	0.0187 (4)	
H17	0.4285	0.3042	0.5430	0.022*	
C18	0.46683 (7)	0.4691 (2)	0.55888 (7)	0.0175 (4)	
H18	0.4439	0.5148	0.5646	0.021*	
C19	0.79370 (7)	0.9131 (2)	0.57507 (7)	0.0164 (4)	
C20	0.83500 (8)	0.9986 (2)	0.57766 (9)	0.0236 (5)	
C21	0.48447 (8)	0.4574 (2)	0.67002 (8)	0.0186 (4)	
H21	0.4615	0.5102	0.6732	0.022*	
C22	0.47493 (8)	0.3288 (2)	0.66318 (8)	0.0210 (5)	
H22	0.4459	0.2952	0.6616	0.025*	
C23	0.50798 (8)	0.2504 (2)	0.65867 (8)	0.0199 (4)	
H23	0.5022	0.1623	0.6540	0.024*	
C24	0.54979 (7)	0.3040 (2)	0.66123 (7)	0.0167 (4)	
H24	0.5734	0.2531	0.6583	0.020*	
C25	0.55664 (7)	0.43257 (19)	0.66811 (7)	0.0132 (4)	
C26	0.60103 (7)	0.49493 (19)	0.67221 (7)	0.0129 (4)	
C27	0.67488 (7)	0.49040 (19)	0.67577 (7)	0.0133 (4)	
C28	0.64464 (7)	0.6745 (2)	0.68549 (7)	0.0131 (4)	
C29	0.64930 (7)	0.81058 (19)	0.69692 (7)	0.0136 (4)	
C30	0.69187 (7)	0.8752 (2)	0.70704 (7)	0.0159 (4)	
H30	0.7179	0.8351	0.7046	0.019*	
C31	0.69508 (8)	1.0001 (2)	0.72074 (7)	0.0175 (4)	
H31	0.7238	1.0470	0.7286	0.021*	
C32	0.65594 (8)	1.0557 (2)	0.72280 (8)	0.0189 (4)	
H32	0.6574	1.1413	0.7321	0.023*	
C33	0.61434 (8)	0.9848 (2)	0.71115 (7)	0.0174 (4)	
H33	0.5874	1.0238	0.7122	0.021*	
C34	0.71538 (7)	0.4187 (2)	0.67460 (7)	0.0139 (4)	
C35	0.71950 (7)	0.2897 (2)	0.68192 (8)	0.0176 (4)	
H35	0.6954	0.2441	0.6859	0.021*	
C36	0.75962 (8)	0.2279 (2)	0.68334 (8)	0.0211 (5)	
H36	0.7635	0.1395	0.6885	0.025*	
C37	0.79361 (8)	0.2975 (2)	0.67704 (8)	0.0194 (4)	
H37	0.8215	0.2581	0.6781	0.023*	
C38	0.78620 (7)	0.4264 (2)	0.66917 (7)	0.0176 (4)	
H38	0.8095	0.4737	0.6644	0.021*	
C39	0.47129 (7)	0.8693 (2)	0.68149 (7)	0.0166 (4)	
C40	0.42931 (8)	0.9569 (2)	0.67623 (9)	0.0248 (5)	
C41	0.84017 (8)	0.9311 (2)	0.71703 (7)	0.0200 (5)	
C42	0.87668 (8)	0.8495 (2)	0.70825 (7)	0.0188 (4)	
C43	0.42845 (8)	0.9852 (2)	0.54474 (8)	0.0195 (4)	
C44	0.39639 (7)	0.8680 (2)	0.53872 (8)	0.0184 (4)	

### Atomic displacement parameters (Å^2^)

**Table:**

	*U*^11^	*U*^22^	*U*^33^	*U*^12^	*U*^13^	*U*^23^
Mn1	0.01286 (15)	0.01297 (16)	0.01736 (16)	−0.00116 (11)	0.00777 (12)	0.00032 (12)
Mn2	0.01176 (15)	0.01322 (16)	0.01684 (16)	0.00182 (11)	0.00761 (12)	0.00042 (12)
F1	0.0330 (8)	0.0321 (8)	0.0386 (9)	−0.0065 (6)	0.0156 (7)	0.0123 (7)
F2	0.0450 (10)	0.0609 (12)	0.0347 (9)	−0.0361 (9)	0.0069 (8)	−0.0051 (8)
F3	0.0386 (9)	0.0346 (10)	0.145 (2)	−0.0024 (8)	0.0648 (13)	−0.0040 (12)
F4	0.0285 (7)	0.0241 (8)	0.0556 (10)	0.0101 (6)	0.0218 (7)	0.0173 (7)
F5	0.0209 (7)	0.0416 (10)	0.0715 (12)	0.0082 (7)	0.0270 (8)	0.0209 (9)
F6	0.0592 (12)	0.0725 (14)	0.0429 (10)	0.0348 (10)	0.0235 (9)	−0.0124 (10)
F7	0.0283 (7)	0.0452 (9)	0.0190 (7)	0.0066 (7)	0.0090 (6)	−0.0082 (6)
F8	0.0216 (7)	0.0384 (9)	0.0543 (10)	−0.0090 (6)	0.0220 (7)	−0.0190 (8)
F9	0.0607 (11)	0.0324 (9)	0.0480 (10)	0.0160 (8)	0.0356 (9)	0.0190 (8)
F10	0.0317 (7)	0.0330 (8)	0.0259 (7)	−0.0086 (6)	0.0132 (6)	0.0049 (6)
F11	0.0145 (6)	0.0263 (7)	0.0316 (8)	−0.0023 (5)	0.0058 (6)	−0.0007 (6)
F12	0.0287 (7)	0.0239 (7)	0.0409 (9)	0.0011 (6)	0.0148 (7)	−0.0113 (6)
O1	0.0176 (7)	0.0198 (8)	0.0307 (9)	−0.0006 (6)	0.0138 (7)	−0.0018 (7)
O2	0.0185 (7)	0.0175 (8)	0.0221 (8)	−0.0014 (6)	0.0095 (6)	−0.0010 (6)
O3	0.0236 (8)	0.0174 (8)	0.0267 (9)	0.0045 (6)	0.0150 (7)	0.0042 (6)
O4	0.0155 (7)	0.0226 (8)	0.0284 (9)	0.0011 (6)	0.0122 (7)	−0.0031 (7)
O5	0.0254 (9)	0.0425 (11)	0.0379 (10)	−0.0188 (8)	0.0215 (8)	−0.0205 (9)
O6	0.0170 (7)	0.0219 (8)	0.0271 (9)	−0.0010 (6)	0.0071 (7)	−0.0042 (7)
O7	0.0144 (7)	0.0241 (9)	0.0419 (10)	0.0025 (6)	0.0098 (7)	0.0115 (8)
O8	0.0181 (8)	0.0176 (8)	0.0408 (10)	0.0017 (6)	0.0090 (7)	0.0052 (7)
O1w	0.0246 (8)	0.0155 (8)	0.0189 (8)	−0.0056 (6)	0.0059 (7)	0.0012 (6)
O2w	0.0179 (7)	0.0260 (9)	0.0188 (8)	0.0067 (6)	0.0086 (6)	0.0036 (7)
O3w	0.0200 (7)	0.0139 (7)	0.0177 (8)	0.0051 (6)	0.0077 (6)	0.0013 (6)
O4w	0.0237 (8)	0.0217 (8)	0.0193 (8)	0.0076 (6)	0.0112 (7)	0.0025 (6)
N1	0.0147 (8)	0.0129 (8)	0.0159 (9)	−0.0017 (6)	0.0074 (7)	−0.0004 (7)
N2	0.0137 (8)	0.0124 (8)	0.0157 (8)	−0.0002 (6)	0.0073 (7)	−0.0001 (7)
N3	0.0165 (8)	0.0151 (9)	0.0166 (9)	−0.0016 (7)	0.0090 (7)	−0.0007 (7)
N4	0.0132 (8)	0.0141 (9)	0.0170 (9)	−0.0008 (6)	0.0074 (7)	−0.0008 (7)
N5	0.0122 (8)	0.0138 (8)	0.0161 (9)	−0.0003 (6)	0.0066 (7)	0.0001 (7)
N6	0.0129 (8)	0.0165 (9)	0.0162 (9)	−0.0002 (7)	0.0069 (7)	−0.0006 (7)
N7	0.0131 (8)	0.0147 (8)	0.0156 (8)	0.0008 (7)	0.0070 (7)	0.0001 (7)
N8	0.0123 (8)	0.0128 (8)	0.0151 (8)	−0.0003 (6)	0.0067 (7)	−0.0001 (7)
N9	0.0133 (8)	0.0144 (8)	0.0156 (9)	0.0023 (6)	0.0067 (7)	0.0012 (7)
N10	0.0117 (8)	0.0143 (8)	0.0149 (8)	0.0008 (6)	0.0060 (7)	0.0011 (7)
N11	0.0129 (8)	0.0135 (8)	0.0172 (9)	−0.0001 (6)	0.0074 (7)	0.0012 (7)
N12	0.0127 (8)	0.0155 (9)	0.0182 (9)	0.0006 (7)	0.0076 (7)	0.0013 (7)
C1	0.0174 (10)	0.0141 (10)	0.0237 (11)	−0.0020 (8)	0.0097 (9)	0.0011 (8)
C2	0.0255 (11)	0.0110 (10)	0.0227 (11)	−0.0003 (8)	0.0119 (9)	−0.0009 (8)
C3	0.0211 (10)	0.0173 (11)	0.0246 (11)	0.0025 (8)	0.0130 (9)	−0.0007 (9)
C4	0.0180 (10)	0.0165 (10)	0.0242 (11)	0.0001 (8)	0.0129 (9)	0.0003 (9)
C5	0.0151 (9)	0.0138 (10)	0.0139 (10)	−0.0003 (8)	0.0068 (8)	0.0006 (8)
C6	0.0153 (9)	0.0132 (10)	0.0132 (9)	−0.0015 (7)	0.0062 (8)	−0.0010 (8)
C7	0.0148 (9)	0.0137 (10)	0.0121 (9)	0.0001 (7)	0.0058 (8)	0.0002 (7)
C8	0.0152 (9)	0.0130 (10)	0.0114 (9)	−0.0010 (7)	0.0053 (8)	0.0004 (7)
C9	0.0134 (9)	0.0151 (10)	0.0141 (9)	−0.0008 (7)	0.0073 (8)	−0.0005 (8)
C10	0.0165 (10)	0.0164 (10)	0.0189 (10)	−0.0029 (8)	0.0092 (8)	−0.0019 (8)
C11	0.0214 (11)	0.0141 (10)	0.0257 (12)	−0.0004 (8)	0.0135 (9)	−0.0017 (9)
C12	0.0186 (10)	0.0192 (11)	0.0278 (12)	0.0028 (8)	0.0146 (9)	−0.0002 (9)
C13	0.0161 (10)	0.0195 (11)	0.0259 (11)	−0.0013 (8)	0.0131 (9)	0.0011 (9)
C14	0.0126 (9)	0.0153 (10)	0.0136 (9)	0.0000 (7)	0.0060 (7)	0.0020 (8)
C15	0.0139 (9)	0.0159 (10)	0.0185 (10)	0.0006 (8)	0.0081 (8)	0.0003 (8)
C16	0.0188 (10)	0.0149 (10)	0.0210 (11)	−0.0035 (8)	0.0079 (9)	−0.0004 (8)
C17	0.0127 (9)	0.0207 (11)	0.0208 (11)	−0.0026 (8)	0.0061 (8)	0.0009 (9)
C18	0.0133 (9)	0.0201 (11)	0.0195 (10)	−0.0006 (8)	0.0079 (8)	0.0008 (8)
C19	0.0122 (9)	0.0180 (10)	0.0160 (10)	−0.0020 (8)	0.0038 (8)	0.0022 (8)
C20	0.0156 (10)	0.0212 (11)	0.0335 (13)	−0.0028 (9)	0.0108 (9)	0.0019 (10)
C21	0.0147 (9)	0.0199 (11)	0.0242 (11)	0.0009 (8)	0.0116 (9)	0.0010 (9)
C22	0.0161 (10)	0.0206 (11)	0.0287 (12)	−0.0043 (8)	0.0123 (9)	−0.0010 (9)
C23	0.0203 (11)	0.0148 (10)	0.0255 (12)	−0.0027 (8)	0.0113 (9)	−0.0010 (9)
C24	0.0151 (9)	0.0154 (10)	0.0202 (11)	0.0023 (8)	0.0088 (8)	0.0009 (8)
C25	0.0124 (9)	0.0148 (10)	0.0133 (9)	0.0007 (7)	0.0065 (7)	0.0012 (8)
C26	0.0124 (9)	0.0143 (10)	0.0114 (9)	0.0018 (7)	0.0047 (7)	0.0021 (7)
C27	0.0128 (9)	0.0140 (10)	0.0133 (9)	0.0013 (7)	0.0061 (8)	0.0020 (7)
C28	0.0119 (9)	0.0141 (10)	0.0122 (9)	0.0011 (7)	0.0046 (7)	0.0022 (7)
C29	0.0136 (9)	0.0134 (10)	0.0140 (10)	0.0008 (7)	0.0064 (8)	0.0012 (8)
C30	0.0131 (9)	0.0167 (10)	0.0183 (10)	0.0017 (8)	0.0074 (8)	0.0024 (8)
C31	0.0159 (9)	0.0166 (10)	0.0203 (11)	−0.0033 (8)	0.0085 (8)	0.0007 (8)
C32	0.0227 (11)	0.0120 (10)	0.0223 (11)	−0.0006 (8)	0.0105 (9)	−0.0001 (8)
C33	0.0174 (10)	0.0154 (10)	0.0216 (11)	0.0016 (8)	0.0109 (9)	−0.0004 (8)
C34	0.0114 (9)	0.0161 (10)	0.0134 (9)	0.0003 (7)	0.0051 (7)	−0.0008 (8)
C35	0.0136 (9)	0.0171 (11)	0.0236 (11)	0.0001 (8)	0.0097 (8)	0.0012 (8)
C36	0.0202 (10)	0.0164 (10)	0.0285 (12)	0.0043 (8)	0.0127 (9)	0.0032 (9)
C37	0.0150 (10)	0.0208 (11)	0.0239 (11)	0.0044 (8)	0.0102 (9)	0.0005 (9)
C38	0.0129 (9)	0.0214 (11)	0.0198 (11)	0.0011 (8)	0.0086 (8)	0.0022 (9)
C39	0.0162 (9)	0.0196 (11)	0.0148 (10)	0.0041 (8)	0.0079 (8)	−0.0002 (8)
C40	0.0227 (11)	0.0238 (12)	0.0330 (13)	0.0066 (9)	0.0172 (10)	0.0054 (10)
C41	0.0166 (10)	0.0271 (12)	0.0156 (10)	−0.0036 (9)	0.0068 (8)	−0.0060 (9)
C42	0.0188 (10)	0.0203 (11)	0.0181 (11)	−0.0015 (8)	0.0092 (9)	−0.0007 (8)
C43	0.0181 (10)	0.0186 (11)	0.0226 (11)	−0.0009 (8)	0.0098 (9)	0.0029 (9)
C44	0.0163 (10)	0.0183 (11)	0.0210 (11)	0.0010 (8)	0.0087 (9)	−0.0006 (9)

### Geometric parameters (Å, °)

**Table:**

Mn1—O1	2.301 (2)	N11—C28	1.330 (3)
Mn1—O2	2.396 (2)	N11—C27	1.341 (3)
Mn1—O1w	2.119 (2)	N12—C38	1.335 (3)
Mn1—O2w	2.146 (2)	N12—C34	1.345 (3)
Mn1—N1	2.389 (2)	C1—C2	1.391 (3)
Mn1—N2	2.261 (2)	C1—H1	0.9500
Mn1—N3	2.411 (2)	C2—C3	1.384 (3)
Mn2—O3	2.440 (2)	C2—H2	0.9500
Mn2—O4	2.293 (2)	C3—C4	1.383 (3)
Mn2—O3w	2.127 (2)	C3—H3	0.9500
Mn2—O4w	2.135 (2)	C4—C5	1.380 (3)
Mn2—N7	2.400 (2)	C4—H4	0.9500
Mn2—N8	2.279 (2)	C5—C6	1.480 (3)
Mn2—N9	2.386 (2)	C7—C14	1.485 (3)
F1—C20	1.330 (3)	C8—C9	1.480 (3)
F2—C20	1.324 (3)	C9—C10	1.384 (3)
F3—C20	1.316 (3)	C10—C11	1.389 (3)
F4—C40	1.330 (3)	C10—H10	0.9500
F5—C40	1.340 (3)	C11—C12	1.382 (3)
F6—C40	1.312 (3)	C11—H11	0.9500
F7—C42	1.333 (3)	C12—C13	1.392 (3)
F8—C42	1.334 (3)	C12—H12	0.9500
F9—C42	1.330 (3)	C13—H13	0.9500
F10—C44	1.337 (3)	C14—C15	1.386 (3)
F11—C44	1.339 (2)	C15—C16	1.389 (3)
F12—C44	1.348 (3)	C15—H15	0.9500
O1—C19	1.240 (3)	C16—C17	1.384 (3)
O2—C19	1.248 (3)	C16—H16	0.9500
O3—C39	1.252 (3)	C17—C18	1.388 (3)
O4—C39	1.235 (3)	C17—H17	0.9500
O5—C41	1.244 (3)	C18—H18	0.9500
O6—C41	1.232 (3)	C19—C20	1.542 (3)
O7—C43	1.238 (3)	C21—C22	1.390 (3)
O8—C43	1.236 (3)	C21—H21	0.9500
O1w—H1w1	0.84 (3)	C22—C23	1.381 (3)
O1w—H1w2	0.84 (3)	C22—H22	0.9500
O2w—H2w1	0.84 (3)	C23—C24	1.387 (3)
O2w—H2w2	0.84 (3)	C23—H23	0.9500
O3w—H3w1	0.84 (3)	C24—C25	1.380 (3)
O3w—H3w2	0.84 (3)	C24—H24	0.9500
O4w—H4w1	0.84 (3)	C25—C26	1.481 (3)
O4w—H4w2	0.84 (3)	C27—C34	1.486 (3)
N1—C1	1.332 (3)	C28—C29	1.479 (3)
N1—C5	1.347 (3)	C29—C30	1.390 (3)
N2—C6	1.328 (3)	C30—C31	1.387 (3)
N2—C8	1.341 (3)	C30—H30	0.9500
N3—C13	1.331 (3)	C31—C32	1.382 (3)
N3—C9	1.352 (3)	C31—H31	0.9500
N4—C6	1.333 (3)	C32—C33	1.391 (3)
N4—C7	1.338 (3)	C32—H32	0.9500
N5—C8	1.331 (3)	C33—H33	0.9500
N5—C7	1.341 (3)	C34—C35	1.384 (3)
N6—C18	1.333 (3)	C35—C36	1.391 (3)
N6—C14	1.346 (3)	C35—H35	0.9500
N7—C21	1.336 (3)	C36—C37	1.380 (3)
N7—C25	1.349 (3)	C36—H36	0.9500
N8—C26	1.333 (3)	C37—C38	1.388 (3)
N8—C28	1.333 (3)	C37—H37	0.9500
N9—C33	1.336 (3)	C38—H38	0.9500
N9—C29	1.344 (3)	C39—C40	1.546 (3)
N10—C26	1.335 (3)	C41—C42	1.556 (3)
N10—C27	1.337 (3)	C43—C44	1.548 (3)
			
O1w—Mn1—O2w	175.89 (6)	C12—C11—H11	120.8
O1w—Mn1—N2	86.97 (6)	C10—C11—H11	120.8
O2w—Mn1—N2	88.92 (6)	C11—C12—C13	119.1 (2)
O1w—Mn1—O1	90.24 (6)	C11—C12—H12	120.4
O2w—Mn1—O1	93.43 (6)	C13—C12—H12	120.4
N2—Mn1—O1	152.20 (6)	N3—C13—C12	123.3 (2)
O1w—Mn1—N1	90.44 (6)	N3—C13—H13	118.4
O2w—Mn1—N1	88.19 (6)	C12—C13—H13	118.4
N1—Mn1—N2	69.67 (6)	N6—C14—C15	123.30 (19)
O1—Mn1—N1	82.71 (6)	N6—C14—C7	115.48 (18)
O1w—Mn1—O2	98.86 (6)	C15—C14—C7	121.21 (18)
O2w—Mn1—O2	84.74 (6)	C14—C15—C16	118.39 (19)
N2—Mn1—O2	151.37 (6)	C14—C15—H15	120.8
O1—Mn1—O2	56.30 (5)	C16—C15—H15	120.8
N1—Mn1—O2	137.72 (6)	C17—C16—C15	118.9 (2)
O1w—Mn1—N3	84.67 (6)	C17—C16—H16	120.6
O2w—Mn1—N3	93.79 (6)	C15—C16—H16	120.6
N1—Mn1—N3	138.46 (6)	C16—C17—C18	118.6 (2)
N2—Mn1—N3	68.89 (6)	C16—C17—H17	120.7
O1—Mn1—N3	138.36 (6)	C18—C17—H17	120.7
O2—Mn1—N3	83.67 (6)	N6—C18—C17	123.5 (2)
O3w—Mn2—O4w	176.32 (7)	N6—C18—H18	118.3
O3w—Mn2—N8	87.81 (6)	C17—C18—H18	118.3
O4w—Mn2—N8	90.92 (6)	O1—C19—O2	126.0 (2)
O3w—Mn2—O4	82.22 (6)	O1—C19—C20	115.96 (19)
O4w—Mn2—O4	100.56 (6)	O2—C19—C20	118.01 (19)
N8—Mn2—O4	149.21 (6)	F3—C20—F2	107.1 (2)
O3w—Mn2—N9	95.84 (6)	F3—C20—F1	107.7 (2)
O4w—Mn2—N9	86.93 (6)	F2—C20—F1	106.6 (2)
N7—Mn2—N8	68.46 (6)	F3—C20—C19	112.7 (2)
N7—Mn2—N9	137.33 (6)	F2—C20—C19	111.9 (2)
N8—Mn2—N9	69.20 (6)	F1—C20—C19	110.60 (18)
O4—Mn2—N9	82.87 (6)	N7—C21—C22	122.9 (2)
O3w—Mn2—N7	87.62 (6)	N7—C21—H21	118.5
O4w—Mn2—N7	88.70 (6)	C22—C21—H21	118.5
O4—Mn2—N7	139.49 (6)	C23—C22—C21	119.4 (2)
O3w—Mn2—O3	94.37 (6)	C23—C22—H22	120.3
O4w—Mn2—O3	85.30 (6)	C21—C22—H22	120.3
N8—Mn2—O3	154.58 (6)	C22—C23—C24	118.1 (2)
O4—Mn2—O3	55.78 (5)	C22—C23—H23	120.9
N9—Mn2—O3	135.41 (6)	C24—C23—H23	120.9
N7—Mn2—O3	86.31 (6)	C25—C24—C23	119.0 (2)
C19—O1—Mn1	91.12 (13)	C25—C24—H24	120.5
C19—O2—Mn1	86.56 (12)	C23—C24—H24	120.5
C39—O3—Mn2	85.32 (12)	N7—C25—C24	123.45 (19)
C39—O4—Mn2	92.45 (13)	N7—C25—C26	115.02 (18)
Mn1—O1w—H1w1	129.2 (17)	C24—C25—C26	121.51 (18)
Mn1—O1w—H1w2	119.8 (17)	N8—C26—N10	124.61 (18)
H1w1—O1w—H1w2	110.4 (16)	N8—C26—C25	115.88 (17)
Mn1—O2w—H2w1	121.8 (17)	N10—C26—C25	119.51 (18)
Mn1—O2w—H2w2	124.5 (17)	N10—C27—N11	125.50 (18)
H2w1—O2w—H2w2	109.2 (15)	N10—C27—C34	117.58 (18)
Mn2—O3w—H3w1	122.4 (16)	N11—C27—C34	116.89 (17)
Mn2—O3w—H3w2	127.4 (16)	N11—C28—N8	124.55 (19)
H3w1—O3w—H3w2	110.1 (15)	N11—C28—C29	119.10 (18)
Mn2—O4w—H4w1	126.3 (18)	N8—C28—C29	116.30 (17)
Mn2—O4w—H4w2	116.9 (17)	N9—C29—C30	123.49 (19)
H4w1—O4w—H4w2	109.6 (15)	N9—C29—C28	115.60 (18)
C1—N1—C5	116.85 (18)	C30—C29—C28	120.85 (18)
C1—N1—Mn1	126.24 (14)	C31—C30—C29	117.87 (19)
C5—N1—Mn1	116.90 (13)	C31—C30—H30	121.1
C6—N2—C8	116.00 (18)	C29—C30—H30	121.1
C6—N2—Mn1	121.20 (14)	C32—C31—C30	119.18 (19)
C8—N2—Mn1	122.67 (13)	C32—C31—H31	120.4
C13—N3—C9	117.09 (18)	C30—C31—H31	120.4
C13—N3—Mn1	125.48 (14)	C31—C32—C33	119.1 (2)
C9—N3—Mn1	117.11 (13)	C31—C32—H32	120.4
C6—N4—C7	114.69 (17)	C33—C32—H32	120.4
C8—N5—C7	114.63 (18)	N9—C33—C32	122.46 (19)
C18—N6—C14	117.34 (18)	N9—C33—H33	118.8
C21—N7—C25	117.08 (18)	C32—C33—H33	118.8
C21—N7—Mn2	124.84 (14)	N12—C34—C35	122.91 (19)
C25—N7—Mn2	118.08 (13)	N12—C34—C27	116.23 (18)
C26—N8—C28	115.94 (17)	C35—C34—C27	120.82 (18)
C26—N8—Mn2	122.56 (13)	C34—C35—C36	118.7 (2)
C28—N8—Mn2	121.36 (14)	C34—C35—H35	120.6
C33—N9—C29	117.85 (18)	C36—C35—H35	120.6
C33—N9—Mn2	124.48 (14)	C37—C36—C35	118.8 (2)
C29—N9—Mn2	117.52 (14)	C37—C36—H36	120.6
C26—N10—C27	114.58 (18)	C35—C36—H36	120.6
C28—N11—C27	114.76 (17)	C36—C37—C38	118.6 (2)
C38—N12—C34	117.62 (19)	C36—C37—H37	120.7
N1—C1—C2	123.4 (2)	C38—C37—H37	120.7
N1—C1—H1	118.3	N12—C38—C37	123.3 (2)
C2—C1—H1	118.3	N12—C38—H38	118.3
C3—C2—C1	118.7 (2)	C37—C38—H38	118.3
C3—C2—H2	120.7	O4—C39—O3	126.2 (2)
C1—C2—H2	120.7	O4—C39—C40	116.2 (2)
C4—C3—C2	118.8 (2)	O3—C39—C40	117.59 (19)
C4—C3—H3	120.6	F6—C40—F4	108.5 (2)
C2—C3—H3	120.6	F6—C40—F5	107.8 (2)
C5—C4—C3	118.4 (2)	F4—C40—F5	105.76 (19)
C5—C4—H4	120.8	F6—C40—C39	110.3 (2)
C3—C4—H4	120.8	F4—C40—C39	112.11 (19)
N1—C5—C4	123.83 (19)	F5—C40—C39	112.1 (2)
N1—C5—C6	115.22 (18)	O6—C41—O5	129.4 (2)
C4—C5—C6	120.96 (18)	O6—C41—C42	116.24 (19)
N2—C6—N4	124.64 (19)	O5—C41—C42	114.4 (2)
N2—C6—C5	116.96 (18)	F9—C42—F7	106.66 (19)
N4—C6—C5	118.40 (18)	F9—C42—F8	105.82 (19)
N4—C7—N5	125.51 (19)	F7—C42—F8	106.99 (18)
N4—C7—C14	116.71 (18)	F9—C42—C41	112.29 (18)
N5—C7—C14	117.78 (18)	F7—C42—C41	112.10 (18)
N5—C8—N2	124.42 (19)	F8—C42—C41	112.55 (18)
N5—C8—C9	119.68 (18)	O8—C43—O7	129.6 (2)
N2—C8—C9	115.87 (18)	O8—C43—C44	115.82 (19)
N3—C9—C10	123.41 (19)	O7—C43—C44	114.54 (19)
N3—C9—C8	115.40 (18)	F10—C44—F11	106.79 (17)
C10—C9—C8	121.13 (18)	F10—C44—F12	106.31 (18)
C9—C10—C11	118.67 (19)	F11—C44—F12	106.29 (18)
C9—C10—H10	120.7	F10—C44—C43	111.95 (18)
C11—C10—H10	120.7	F11—C44—C43	113.18 (18)
C12—C11—C10	118.4 (2)	F12—C44—C43	111.86 (18)
			
O1w—Mn1—O1—C19	100.41 (13)	C7—N5—C8—N2	−0.6 (3)
O2w—Mn1—O1—C19	−81.45 (13)	C7—N5—C8—C9	177.49 (18)
N2—Mn1—O1—C19	−175.64 (14)	C6—N2—C8—N5	2.7 (3)
N1—Mn1—O1—C19	−169.19 (13)	Mn1—N2—C8—N5	178.60 (15)
O2—Mn1—O1—C19	−0.09 (12)	C6—N2—C8—C9	−175.49 (17)
N3—Mn1—O1—C19	18.16 (17)	Mn1—N2—C8—C9	0.4 (2)
O1w—Mn1—O2—C19	−84.24 (13)	C13—N3—C9—C10	−0.5 (3)
O2w—Mn1—O2—C19	97.77 (13)	Mn1—N3—C9—C10	−174.35 (16)
N2—Mn1—O2—C19	175.76 (13)	C13—N3—C9—C8	176.72 (18)
O1—Mn1—O2—C19	0.09 (12)	Mn1—N3—C9—C8	2.9 (2)
N1—Mn1—O2—C19	16.29 (16)	N5—C8—C9—N3	179.52 (18)
N3—Mn1—O2—C19	−167.82 (13)	N2—C8—C9—N3	−2.2 (3)
O3w—Mn2—O3—C39	−74.81 (13)	N5—C8—C9—C10	−3.2 (3)
O4w—Mn2—O3—C39	108.87 (13)	N2—C8—C9—C10	175.05 (19)
N8—Mn2—O3—C39	−168.92 (14)	N3—C9—C10—C11	1.1 (3)
O4—Mn2—O3—C39	2.71 (12)	C8—C9—C10—C11	−175.97 (19)
N9—Mn2—O3—C39	28.07 (16)	C9—C10—C11—C12	−0.5 (3)
N7—Mn2—O3—C39	−162.13 (13)	C10—C11—C12—C13	−0.5 (3)
O3w—Mn2—O4—C39	97.98 (13)	C9—N3—C13—C12	−0.7 (3)
O4w—Mn2—O4—C39	−79.58 (13)	Mn1—N3—C13—C12	172.63 (17)
N8—Mn2—O4—C39	170.25 (13)	C11—C12—C13—N3	1.2 (4)
N9—Mn2—O4—C39	−165.10 (13)	C18—N6—C14—C15	0.1 (3)
N7—Mn2—O4—C39	20.95 (17)	C18—N6—C14—C7	179.21 (18)
O3—Mn2—O4—C39	−2.74 (12)	N4—C7—C14—N6	−6.9 (3)
O1w—Mn1—N1—C1	93.77 (18)	N5—C7—C14—N6	173.81 (18)
O2w—Mn1—N1—C1	−90.11 (18)	N4—C7—C14—C15	172.19 (19)
N2—Mn1—N1—C1	−179.62 (19)	N5—C7—C14—C15	−7.1 (3)
O1—Mn1—N1—C1	3.58 (17)	N6—C14—C15—C16	−1.2 (3)
O2—Mn1—N1—C1	−9.9 (2)	C7—C14—C15—C16	179.82 (19)
N3—Mn1—N1—C1	176.22 (16)	C14—C15—C16—C17	1.1 (3)
O1w—Mn1—N1—C5	−87.45 (15)	C15—C16—C17—C18	−0.1 (3)
O2w—Mn1—N1—C5	88.67 (15)	C14—N6—C18—C17	0.9 (3)
N2—Mn1—N1—C5	−0.85 (14)	C16—C17—C18—N6	−0.9 (3)
O1—Mn1—N1—C5	−177.64 (15)	Mn1—O1—C19—O2	0.2 (2)
O2—Mn1—N1—C5	168.83 (13)	Mn1—O1—C19—C20	179.06 (17)
N3—Mn1—N1—C5	−5.01 (19)	Mn1—O2—C19—O1	−0.2 (2)
O1w—Mn1—N2—C6	91.00 (16)	Mn1—O2—C19—C20	−179.03 (18)
O2w—Mn1—N2—C6	−89.06 (16)	O1—C19—C20—F3	170.2 (2)
O1—Mn1—N2—C6	6.2 (2)	O2—C19—C20—F3	−10.8 (3)
N1—Mn1—N2—C6	−0.59 (15)	O1—C19—C20—F2	49.5 (3)
O2—Mn1—N2—C6	−166.02 (14)	O2—C19—C20—F2	−131.5 (2)
N3—Mn1—N2—C6	176.46 (17)	O1—C19—C20—F1	−69.1 (3)
O1w—Mn1—N2—C8	−84.72 (16)	O2—C19—C20—F1	109.8 (2)
O2w—Mn1—N2—C8	95.22 (16)	C25—N7—C21—C22	−0.4 (3)
O1—Mn1—N2—C8	−169.47 (14)	Mn2—N7—C21—C22	179.78 (17)
N1—Mn1—N2—C8	−176.31 (17)	N7—C21—C22—C23	0.2 (4)
O2—Mn1—N2—C8	18.3 (2)	C21—C22—C23—C24	0.0 (3)
N3—Mn1—N2—C8	0.74 (15)	C22—C23—C24—C25	0.0 (3)
O1w—Mn1—N3—C13	−86.45 (18)	C21—N7—C25—C24	0.4 (3)
O2w—Mn1—N3—C13	97.38 (18)	Mn2—N7—C25—C24	−179.75 (16)
N2—Mn1—N3—C13	−175.25 (19)	C21—N7—C25—C26	−178.43 (18)
O1—Mn1—N3—C13	−2.1 (2)	Mn2—N7—C25—C26	1.4 (2)
N1—Mn1—N3—C13	−171.07 (16)	C23—C24—C25—N7	−0.2 (3)
O2—Mn1—N3—C13	13.10 (17)	C23—C24—C25—C26	178.58 (19)
C19—Mn1—N3—C13	6.95 (19)	C28—N8—C26—N10	−3.1 (3)
O1w—Mn1—N3—C9	86.84 (15)	Mn2—N8—C26—N10	−178.80 (15)
O2w—Mn1—N3—C9	−89.33 (15)	C28—N8—C26—C25	175.82 (17)
N2—Mn1—N3—C9	−1.96 (14)	Mn2—N8—C26—C25	0.1 (2)
O1—Mn1—N3—C9	171.19 (13)	C27—N10—C26—N8	1.5 (3)
N1—Mn1—N3—C9	2.22 (19)	C27—N10—C26—C25	−177.44 (18)
O2—Mn1—N3—C9	−173.61 (15)	N7—C25—C26—N8	−1.0 (3)
C19—Mn1—N3—C9	−179.75 (14)	C24—C25—C26—N8	−179.91 (19)
O3w—Mn2—N7—C21	−92.60 (17)	N7—C25—C26—N10	177.98 (18)
O4w—Mn2—N7—C21	87.32 (17)	C24—C25—C26—N10	−0.9 (3)
N8—Mn2—N7—C21	178.82 (19)	C26—N10—C27—N11	0.8 (3)
O4—Mn2—N7—C21	−17.5 (2)	C26—N10—C27—C34	178.67 (17)
N9—Mn2—N7—C21	171.37 (15)	C28—N11—C27—N10	−1.1 (3)
O3—Mn2—N7—C21	1.95 (17)	C28—N11—C27—C34	−179.02 (17)
O3w—Mn2—N7—C25	87.61 (15)	C27—N11—C28—N8	−0.8 (3)
O4w—Mn2—N7—C25	−92.48 (15)	C27—N11—C28—C29	176.68 (18)
N8—Mn2—N7—C25	−0.98 (14)	C26—N8—C28—N11	2.7 (3)
O4—Mn2—N7—C25	162.70 (13)	Mn2—N8—C28—N11	178.47 (15)
N9—Mn2—N7—C25	−8.42 (19)	C26—N8—C28—C29	−174.77 (17)
O3—Mn2—N7—C25	−177.85 (15)	Mn2—N8—C28—C29	1.0 (2)
O3w—Mn2—N8—C26	−87.89 (16)	C33—N9—C29—C30	−0.9 (3)
O4w—Mn2—N8—C26	88.65 (16)	Mn2—N9—C29—C30	−176.60 (16)
O4—Mn2—N8—C26	−158.70 (14)	C33—N9—C29—C28	176.44 (18)
N9—Mn2—N8—C26	175.02 (17)	Mn2—N9—C29—C28	0.7 (2)
N7—Mn2—N8—C26	0.41 (15)	N11—C28—C29—N9	−178.75 (18)
O3—Mn2—N8—C26	7.7 (2)	N8—C28—C29—N9	−1.1 (3)
O3w—Mn2—N8—C28	96.66 (16)	N11—C28—C29—C30	−1.3 (3)
O4w—Mn2—N8—C28	−86.80 (16)	N8—C28—C29—C30	176.31 (19)
O4—Mn2—N8—C28	25.8 (2)	N9—C29—C30—C31	1.9 (3)
N9—Mn2—N8—C28	−0.43 (15)	C28—C29—C30—C31	−175.32 (19)
N7—Mn2—N8—C28	−175.04 (17)	C29—C30—C31—C32	−1.4 (3)
O3—Mn2—N8—C28	−167.76 (14)	C30—C31—C32—C33	0.1 (3)
O3w—Mn2—N9—C33	98.99 (17)	C29—N9—C33—C32	−0.5 (3)
O4w—Mn2—N9—C33	−83.44 (17)	Mn2—N9—C33—C32	174.84 (16)
N8—Mn2—N9—C33	−175.60 (18)	C31—C32—C33—N9	0.9 (3)
O4—Mn2—N9—C33	17.61 (17)	C38—N12—C34—C35	−1.0 (3)
N7—Mn2—N9—C33	−168.19 (15)	C38—N12—C34—C27	176.44 (18)
O3—Mn2—N9—C33	−3.3 (2)	N10—C27—C34—N12	162.14 (18)
O3w—Mn2—N9—C29	−85.62 (15)	N11—C27—C34—N12	−19.8 (3)
O4w—Mn2—N9—C29	91.94 (15)	N10—C27—C34—C35	−20.4 (3)
N8—Mn2—N9—C29	−0.21 (14)	N11—C27—C34—C35	157.7 (2)
O4—Mn2—N9—C29	−167.00 (15)	N12—C34—C35—C36	1.3 (3)
N7—Mn2—N9—C29	7.20 (19)	C27—C34—C35—C36	−176.0 (2)
O3—Mn2—N9—C29	172.08 (13)	C34—C35—C36—C37	−0.5 (3)
C5—N1—C1—C2	−1.3 (3)	C35—C36—C37—C38	−0.5 (3)
Mn1—N1—C1—C2	177.52 (16)	C34—N12—C38—C37	−0.1 (3)
N1—C1—C2—C3	0.6 (3)	C36—C37—C38—N12	0.8 (3)
C1—C2—C3—C4	0.2 (3)	Mn2—O4—C39—O3	5.5 (2)
C2—C3—C4—C5	−0.3 (3)	Mn2—O4—C39—C40	−175.65 (17)
C1—N1—C5—C4	1.1 (3)	Mn2—O3—C39—O4	−5.2 (2)
Mn1—N1—C5—C4	−177.78 (16)	Mn2—O3—C39—C40	175.99 (18)
C1—N1—C5—C6	−179.11 (18)	O4—C39—C40—F6	−84.0 (3)
Mn1—N1—C5—C6	2.0 (2)	O3—C39—C40—F6	94.9 (3)
C3—C4—C5—N1	−0.3 (3)	O4—C39—C40—F4	37.0 (3)
C3—C4—C5—C6	179.9 (2)	O3—C39—C40—F4	−144.0 (2)
C8—N2—C6—N4	−1.8 (3)	O4—C39—C40—F5	155.8 (2)
Mn1—N2—C6—N4	−177.75 (15)	O3—C39—C40—F5	−25.2 (3)
C8—N2—C6—C5	177.83 (18)	O6—C41—C42—F9	130.8 (2)
Mn1—N2—C6—C5	1.8 (2)	O5—C41—C42—F9	−48.3 (3)
C7—N4—C6—N2	−1.1 (3)	O6—C41—C42—F7	−109.1 (2)
C7—N4—C6—C5	179.33 (18)	O5—C41—C42—F7	71.8 (3)
N1—C5—C6—N2	−2.5 (3)	O6—C41—C42—F8	11.5 (3)
C4—C5—C6—N2	177.27 (19)	O5—C41—C42—F8	−167.6 (2)
N1—C5—C6—N4	177.10 (18)	O8—C43—C44—F10	−110.1 (2)
C4—C5—C6—N4	−3.1 (3)	O7—C43—C44—F10	69.7 (3)
C6—N4—C7—N5	3.5 (3)	O8—C43—C44—F11	10.7 (3)
C6—N4—C7—C14	−175.73 (17)	O7—C43—C44—F11	−169.6 (2)
C8—N5—C7—N4	−2.7 (3)	O8—C43—C44—F12	130.7 (2)
C8—N5—C7—C14	176.52 (17)	O7—C43—C44—F12	−49.5 (3)

### Hydrogen-bond geometry (Å, °)

**Table:**

*D*—H···*A*	*D*—H	H···*A*	*D*···*A*	*D*—H···*A*
O1w—H1w1···O5	0.84 (3)	1.82 (3)	2.661 (2)	176 (3)
O1w—H1w2···N12	0.84 (3)	1.94 (3)	2.765 (2)	166 (3)
O2w—H2w1···O2^i^	0.84 (3)	1.96 (3)	2.789 (2)	169 (3)
O2w—H2w2···O8^ii^	0.84 (3)	1.85 (3)	2.679 (2)	167 (3)
O3w—H3w1···N6	0.84 (3)	1.98 (3)	2.798 (2)	167 (2)
O3w—H3w2···O7	0.84 (3)	1.86 (3)	2.703 (2)	175 (3)
O4w—H4w1···O3^iii^	0.84 (3)	1.95 (3)	2.782 (2)	170 (3)
O4w—H4w2···O6^iv^	0.84 (3)	1.87 (3)	2.672 (2)	160 (2)

Symmetry codes: (i) −*x*+3/2, −*y*+3/2, −*z*+1; (ii) −*x*+1, −*y*+2, −*z*+1; (iii) −*x*+1, *y*, −*z*+3/2; (iv) −*x*+3/2, *y*−1/2, −*z*+3/2.
